# Towards Solving Health Inequities: A Method to Identify Ideological Operation in Global Health Programs

**DOI:** 10.3390/ijerph18094393

**Published:** 2021-04-21

**Authors:** Hani Kim, Uros Novakovic

**Affiliations:** 1 Bill & Melinda Gates Foundation 500 5th Ave North, Seattle, WA 98109, USA; 2 Office Ou, Toronto, ON M6E 3H3, Canada; uros@office-ou.com

**Keywords:** ideology, bias, inequality, power, Slavoj Žižek, antimicrobial resistance, race, vaccine

## Abstract

The function of ideology is to naturalize and maintain unequal relations of power. Making visible how ideology operates is necessary for solving health inequities grounded in inequities of resources and power. However, discerning ideology is difficult because it operates implicitly. It is not necessarily explicit in one’s stated aims or beliefs. Philosopher Slavoj Žižek conceptualizes ideology as a belief in overarching unity or harmony that obfuscates immanent tension within a system. Drawing from Žižek’s conceptualization of ideology, we identify what may be considered as ‘symptoms’ of ideological practice: (1) the recurrent nature of a problem, and (2) the implicit externalization of the cause. Our aim is to illustrate a method to identify ideological operation in health programs on the basis of its symptoms, using three case studies of persistent global health problems: inequitable access to vaccines, antimicrobial resistance, and health inequities across racialized communities. Our proposed approach for identifying ideology allows one to identify ideological practices that could not be identified by particular ideological contents. It also safeguards us from an illusory search for an emancipatory content. Critiquing ideology in general reveals possibilities that are otherwise kept invisible and unimaginable, and may help us solve recalcitrant problems such as health inequities.

## 1. Introduction

Health inequities are commonly rooted in inequalities in material conditions, which determine what individuals can do to ensure their survival and flourishing. These inequalities are produced within unequal relations of power. Naturalizing and legitimizing unequal power relations is the function of ideology. Making visible and contesting ideology is necessary for solving root causes of health inequities. However, discerning ideology is difficult and may sometimes be seen as impossible. The difficulty arises because ideology operates implicitly, and not necessarily through explicitly stated intent or beliefs.

Ideologies are matrices of meaning and normativity, but we should not expect to find them laid out in convenient formulas. They are usually implicit rather than explicit, the underlying logic that facilitates the move from premise to conclusion in the act of political reasoning. And what makes most sense is precisely what needs to be affirmed the least, as it can be taken for granted. Ideology must be excavated; it will not necessarily be written in letters ten feet tall [1].

Our objective in this paper is two-fold: first, to propose symptoms of ideological operation grounded in the conceptualization of ideology by philosopher Slavoj Žižek, and second, to illustrate a systematic approach to identify ideological operation in health research and programs on the basis of these symptoms.

### 1.1. Ideological Bias in Science

The pernicious effects of bias on science have been well recognized by both scientists and by the public. Bias is known to affect selection, conduct, and reporting in scientific studies [2,3,4,5]. Scientists have been striving to detect, explicate, and critically examine different types of biases in an effort to make science “objective, transparent, and free from bias” [6]. Ideological bias may be considered a type of bias that, in contrast to biases from personal preferences (e.g., for red wine over white wine), operates throughout an entire field or a society, and serves particular power interests [7,8]. Ideology serves as a matrix through which power governs reasoning and assigns hierarchy to knowledge, values, and beliefs [7,8].

In health research, the term ‘ideology’ has been used interchangeably with particular values or belief systems such as ‘neoliberalism’, ‘racism’, ‘colonialism’, or ‘patriarchy’ that legitimize inequalities [9,10,11,12,13,14,15]. For example, critiques of racism highlight racialization as a social mechanism to legitimize inequalities [16]. The racialized global health practice in the 19th century was legitimized as a moral duty of the colonizing nations to ‘civilize’ and ‘save’ the ‘primitive’ based on an assumed sense of racial, intellectual, or cultural superiority [17,18]. Similarly, critiques of neoliberalism highlight the dominant norm of prioritizing individual merits and entrepreneurship (over collective actions) and markets (over governments), and meritocracy as a narrative to legitimize economic inequalities [14,19]. In global health programs, neoliberal values individualize and depoliticize causes of and solutions to ill health, prioritize individual-level factors (i.e., biological, behavioral) as desirable targets of interventions [20], and emphasize technological solutions [20,21,22].

These critiques targeting particular beliefs or values (ideological content) are valuable in illuminating narratives that legitimize inequalities in material conditions and power. However, limiting ideological critiques to critiques of particular contents is inadequate in a number of ways. First, it is predicated on an already identifiable ideological content. For example, an anti-racist or anti-patriarchy stance requires racism or patriarchy to be already recognized. Critiquing ideological content is necessarily reactive, and risks losing sight of new narratives that are still *in the making* to legitimize inequalities. Second, it has been observed that the proliferation of critiques (i.e., production of knowledge) does not necessarily translate into a transformative change, and this ‘critical knowledge’ may, in fact, be susceptible to being appropriated to legitimize inequalities in material conditions and power [23].

In his work, Slavoj Žižek provides a possible framework for critiquing ideology *in general* that is independent of particular ideological content. Žižek reinterprets the notion of ideology as a product of subjects’ failure to confront an irreducible gap between the subjects and symbolic representation. According to Žižek, we ‘ideologically’ interpret the social world in our Sysiphian effort to fill the gap [24].

Subjectivization designates the movement through which the subject integrates what is given them into the universe of meaning—integration always ultimately fails, there is a certain *left-over which cannot be integrated into the symbolic universe, an object which resists subjectivization*, and the subject is precisely correlative to this object. In other words, the subject is correlative to its own limit, to the element which cannot be subjectivized, it is the name of the void which cannot be filled out with subjectivization: the subject is the point of failure of subjectivization (emphasis by the authors) [25].

Ideology need not be imposed onto individuals by a ruling class or state apparatus. Individuals actively produce and reproduce ideology through ritualized daily practices in our futile attempt to fill this pre-transcendental ‘void’.

The work of Žižek is useful because, first, it provides a formal conceptualization of ideology in general as an effort to fill the gap. Second, it makes ideology as a form observable by locating it in the implicit in the texture of our daily life rather than in explicit beliefs or particular knowledge; this is of significant importance in suggesting a new mode of political struggle and resistance. Žižek’s conceptualization of ideology provides a theoretical framework for moving beyond the critique of ideological content to a critique of ideological operation that can be identified in health programs and research.

### 1.2. Ideology as a Belief in Overarching Unity and Harmony

As conceptualized by Žižek, ideology performs its function of naturalizing unequal relations of power by portraying a system capable of regulating itself towards harmony and unity, devoid of inner tension [24,25,26] It belies an implicit belief in a higher order that redeems any social conflicts and ultimately guarantees unity and harmony within the existing social order [26]. In an ideological conceptual matrix, social conflicts and problems are portrayed as if they are rooted in something extrinsic, rather than constitutive of the social structure itself.

For example, neoliberal ideology imagines an unregulated market economy as self-stabilizing (e.g., as in ‘trickle-down’ economics) and views the cause of a social problem such as extreme concentration of wealth as a malfunctioning of the market rather than its inherent property. Similarly, racist, patriarchal, or colonialist ideologies imagine an inherent harmony in the hierarchy of different racial, gender, or cultural groups. Social conflicts are portrayed as if they are caused by resistance to this otherwise harmonious natural social order. Indeed, the dominant narrative portrays the ideology critics as external intruders (e.g., ‘rebel rousers’) who disturb a system that is working its way towards harmony. By portraying inherent harmony and obscuring the inherent tension, ideology keeps the inner workings of power hidden from view and critical inquiries. It forecloses critiques of the unequal and unjust power structure itself as the very source of social inequalities.

According to Žižek, what makes something ideological is not this or that idea or belief per se, but an invisible matrix that obscures the inner necessity of conflicts in a society. Conceptualized this way, his critique of ideology assumes no inherently ‘emancipatory’ or ‘non-ideological’ content. An emancipatory potential lies in making visible the immanent tension [24,27].

### 1.3. Ideological Operation through the Implicit

For Žižek, ideological operation may be observed in what is implicit in the doing (i.e., meta rules, customs, and habits) of our everyday life, and not through one’s explicitly stated thoughts or beliefs [7,8]. For example, individuals know very well that money is an expression of social relations, and that there is nothing inherent about money that makes it the immediate embodiment of wealth [28]. There is no false recognition of what money really is. Yet, individuals act as if the embodiment of wealth is a natural property of money. Their actions and relations around money are guided by a fetishistic illusion about money during commodity exchanges [29]. In other words, what structures social reality around money is not individuals’ distorted understanding of what money really is (explicit knowledge), but through what is implicit in how people treat money. This is the level at which ideology operates.

Following Žižek’s conceptualization of ideological operation, a barrier to solving social problems like health inequities does not necessarily lie in explicitly stated beliefs or knowledge, but in the misrecognition of what structures social reality. It is theimplicit in what we do in our daily life, not the explicitly articulated ideas or beliefs, that legitimizes and naturalizes the prevailing social order.

At the implicit level, the overarching belief in inherent harmony is associated with two affective dimensions: comfort, relief, or redemption from the assurance of harmony and, conversely, fear of having the harmony threatened by external intruders. The two affective dimensions represent a symptom of ideological operation on one hand, and the mechanism through which ideology operateson the other hand. The sense of fear serves to externalize causes of social inequalities by implicitly denying their root within the system itself (e.g., police brutality against Black Americans as a result of a few ‘bad apples’ among the police officers rather than racism entrenched in the system). As a mechanism for ensuring recurrent production of problems, a sense of comfort assumes an external guarantor of a higher order that would eventually restore social harmony, and it exempts individuals from confronting root causes of problems within the social structure itself. As such, root causes inherent in the social structure evade scrutiny and problems recur.

### 1.4. Symptoms of Ideology

By portraying the social system as capable of regulating itself towards harmony and unity, ideological operation obfuscates the social tension that is inherent within the system, and results in several consequences that may be identified as its symptoms.

First, ideological operation forecloses critically examining the social structure itself as a source of problems. As a result, it limits our ability to observe and solve the root causes of social problems, and ensures that the problems recur in various disguises, as recently conceptualized by a balloon compressed in one corner expanding in another in response to different health interventions (recurrent nature) [30].

Second, as ideology assumes inherent harmony in a social structure, it implicitly portrays social problems as a result of a threat from outside the social structure and externalizes the causes of social problems (implicit externalization of the cause of the problem). Accordingly, an ideological procedure assumes and seeks solutions that lie within the logic of the system.

In this paper, we propose a method to identify ideological operation in health programs on the basis of the symptoms of ideological operation drawn from Žižek’s conceptualization of ideology. We illustrate our method in three case studies of global health problems: (1) inequities in access to vaccines, (2) antimicrobial resistance, and (3) health inequities across racialized communities. Specifically, we describe the recurrent nature of the problem and the externalization of the causes of each of the three health problems.

## 2. Case Studies: Symptoms of Ideological Operation in Global Health Programs

### 2.1. Case Study 1: Global Inequities in Access to Vaccines

#### 2.1.1. Recurrent Nature of the Problem

Global inequity in access to vaccines as an essential medicine has been recognized as a public health problem since as early as the late 1990s, when routine immunization coverage was observed to be highly unequal across and within countries. The low- and middle-income countries (LMICs) have been consistently reported to bear a disproportionate burden of vaccine-preventable diseases (VPDs). The disproportionate burden of VPDs is associated with a gap in the immunization coverage. While UNICEF declared the universal child immunization goal of 80% of the target was achieved in 1990, this aggregate indicator reflected the high coverage rates achieved in some relatively big countries and did not reflect that universal coverage was not reached in 107 countries or that national success conceals poor coverage rates in marginalized populations [31,32].

The problem of inequity in vaccine access is not limited to LMICs. A report from 2003 recognized the problem of high prices of new vaccines and persistent inequalities in immunization levels in the USA [33]. Importantly, the report highlighted the limitations of the public–private partnership model of purchasing and distributing vaccines in the U.S. for the previous 50 years [33].

A proposed solution was a new procuring mechanism to negotiate lower vaccine prices with manufacturers for LMICs in return for a “long-term, high-volume, and predictable demand from those countries” [34]. This gave rise to Gavi the Vaccine Alliance in 2000: a public–private partnership that pools funds, projects the global demand and supply of vaccines deemed essential for child health, and negotiates an affordable price with commercial vaccine manufacturers in exchange for a guaranteed profit through a mechanism known as advanced market commitment (AMC) [31,34,35]. The AMC mechanism provides financial incentives to commercial vaccine manufacturers for diseases for which there is little or no profit.

**Figure 1:**
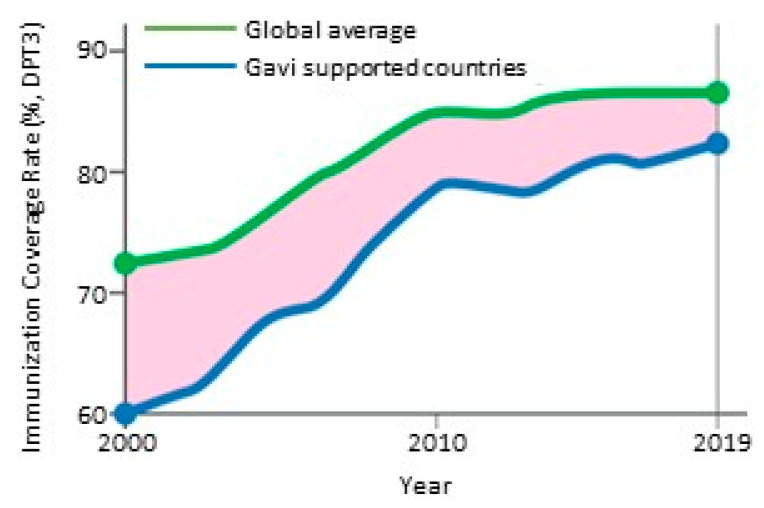
Contribution of Gavi in childhood immunization [36]. Coverage with third dose of diphtheria-pertussis-tetanus-containing vaccine. Source: WHO/UNICEF Estimates of National Immunization Coverage, 2020. Figure adapted from Gavi Annual Progress Report [36].

The Gavi AMC mechanism has significantly contributed to lowering the price of vaccines, and to immunizing children with vaccines that were previously unaffordable (Figure 1) [36]. Nonetheless, the problem of vaccine affordability and inequitable access to vaccines remain. Concerns with the long-term sustainability of the Gavi AMC mechanism have been raised since its inception but remain unresolved [35,37,38]. First, affordable price is determined largely by unverifiable production costs claimed by commercial vaccine manufacturers, rather than the ability of governments to pay [35,37,38]. Second, restrictive licensing and intellectual property regulations prevent the transfer of technologies to small vaccine manufacturers [35,37,38]. They hinder LMICs from developing their own vaccines, and foster domination of vaccine supply by multinational pharmaceutical companies in the high-income countries(HICs) [39]. A Médecins Sans Frontières (MSF) report from 2017 emphasized the persistent problem of the prohibitively high vaccine prices that pose a barrier to many countries to fully immunize a child [39]. The report further highlights the barrier posed by intellectual patents to timely development of and access to affordable versions of newer vaccines. A 2020 UNICEF report concluded that countries, whether receiving support through Gavi or self-financing, continue to face challenges in securing financing/funding to support their immunization programs [40].

Twenty years after Gavi was established, the problem of inequitable access to vaccines has returned amidst the COVID-19 pandemic. Modelled after Gavi AMC, COVAX was established as a mechanism to ensure equitable access to COVID-19 vaccines [41]. Familiar questions were raised regarding how the vaccine doses will be allocated across countries according to the needs, and not the ability to pay, and the possibility of royalty-free tech transfer to multiple manufacturers [42,43]. These questions remain unresolved.

#### 2.1.2. Implicit Externalization of the Cause

The problem of global inequalities in immunization has been portrayed as a result of a market failure [34]. Implicit in this framing is that first, if a market did not fail, it would regulate itself to ensure that the price of vaccines would be adjusted according to the needs of a population regardless of its ability to pay. This implicit assumption contradicts the fundamental rules of commodity exchange in a market economy. The narrative of market failure obscures the inner necessity of inequity in vaccine access within the market-based production and exchange of vaccines.

Similarly, concentration of resources within, and thus, monopoly on the production and ownership of vaccines by large commercial manufacturers in HICs a predictable consequence, not an accident, of the current global intellectual property framework that undergirds the global commercial production and exchange of vaccines as commodities. By implicitly locating the cause of inequitable access to vaccines outside the rules of the market, this narrative precludes the inner workings of the market itself as a source of the problem. As a result, the commercial production and ownership of vaccines are naturalized as if they are something immutable. Since 2000, strategies to ensure equitable access to vaccines have been largely limited to negotiating with commercial vaccine manufacturers to lower the price in exchange for a guaranteed profit. The limited observation into the potential root causes of the problem may underlie the limited strategies and the recurrent nature of the problem.

#### 2.1.3. Summary

The limited strategies, and ultimately, recurrent failure to solve inequitable access to vaccines can be interpreted as a symptom of an ideological practice. The dominant narrative implicitly externalizes the causes of the problem as a market failure and forecloses critical inquiries into the inherent contradiction in the desire to provide vaccines as a public good within the rules of commodity exchange. Externalizing the cause obscures the immanent nature of the problem within the current system of production and ownership of vaccines as commodities. Despite the legitimate contribution of Gavi and its AMC mechanism to improving equitable access to vaccines, this inherent contradiction remains, and yet evades critical inquiries and may limit our action. The problem of financing vaccines for equitable access occurs as a recurrent failure, as we are witnessing with the provision of COVID-19 vaccines.

### 2.2. Case Study 2: Antimicrobial Resistance (AMR)

#### 2.2.1. Recurrent Nature of the Problem

**Figure 2:**
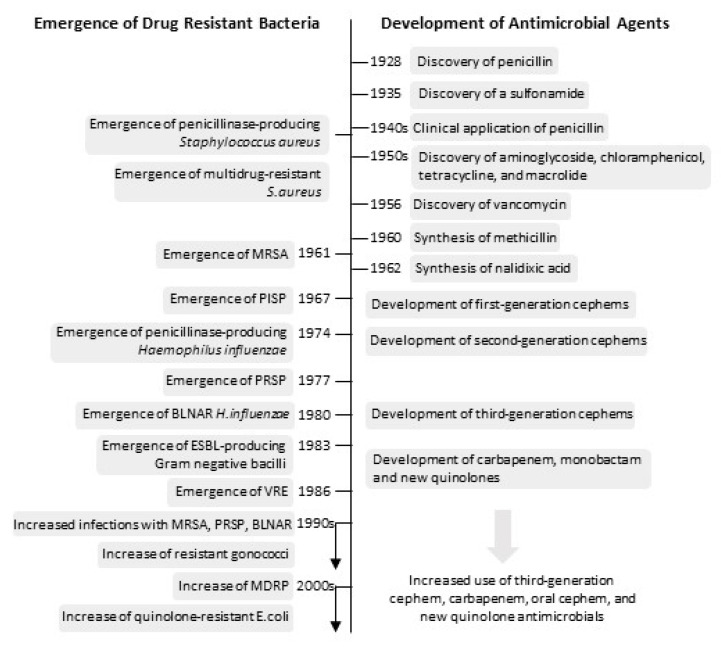
Trend of development of antimicrobial agents and emergence of drug-resistant bacteria [46]. MRSA, Methicillin-resistant Staphylococcus aureus; PISP, penicillin-intermediate-resistant S. pneumoniae; PRSP, penicillin-resistant S. pneumoniae; VRE, vancomycin-resistant enterococci; BLNAR, β-lactamase-negative ampicillin-resistant; MDRP, Multidrug-Resistant Pseudomonas Aeruginosa. Figure adapted from [46].

Antimicrobial resistance(AMR) is the resistance of microorganisms, such as bacteria, viruses, fungi, and parasites, to the killing effects of antimicrobial agents [44]. In early 1945, soon after Alexander Fleming’s discovery of penicillin in 1928, he predicted that the high public demand for antibiotics would determine an “era of abuse”; this eventually became a reality [45]. The history of AMR mirrors the history of antimicrobial agents (Figure 2) [45,46]. The wide clinical application of every class of antimicrobial agents was soon followed by the emergence of bacterial strains that have developed adaptive mutations to evade the microbicidal effects of the drug. Development of the first effective antimicrobial, sulfonamides in 1937, was soon followed by sulfonamide resistance in the late 1930s [46,47]. The first cases of penicillin resistance were documented in 1942 [45]. Similarly, since the first documented case of drug-resistant tuberculosis against streptomycin in 1948 [48], the advent of every new class of drugs against TB led to the emergence of a new drug-resistant strain of TB; a cause for significant concern at a global scale [49]. AMR is not limited to bacteria. Following the detection of the first case of the drug-resistant Plasmodium parasite, a causative agent of malaria, against quinine in 1910 [50], every class of antimalarial drug led to the emergence of new strains that were resistant against the new drugs, including the latest class of antimalarial drug, artemisinin [51]. After more than a century since the first case of antimicrobial resistance, the race continues between the discovery of new antimicrobial agents and the emergence of newly resistant microbes. AMR remains unsolved as a global problem.

#### 2.2.2. Implicit Externalization of the Cause

Recognizing AMR as a global problem with a growing sense of urgency, the One Health framework locates the problem of AMR within the food chain, linking human consumption, agricultural use, and contamination of the environment (e.g., water, soil) [52,53,54]. In this frame, AMR is posited as an anthropogenic problem: a natural adaptive response of microbes to the selection pressure exerted by human activities around the production and use of antimicrobials in health care and agriculture [53,55,56].

Despite what the One Health framework explicitly acknowledges, the implicit in the dominant policy portrays the root cause of AMR as microbes as an external threat, detached from the political economy of the human activities surrounding the production and use of antibiotics. The current policy responses tend to disproportionately focus on two strategies: (1) change the behavior of those who prescribe and those who ask for antimicrobials, and (2) develop new antimicrobials or alternatives to microbials (e.g., vaccines, therapeutic antibodies) [53,57,58]. In the first strategy, the causes of AMR are depoliticized to individual-level knowledge or attitude, as if individuals’ supposed choice to use antimicrobials appropriately can be detached from the sociopolitical context. For example, individuals’ choice to use antibiotics cannot be detached from the lack of access to other alternatives to prevent and control infectious diseases (e.g., nutrition, hygiene, sanitation), and the relatively easy access to antimicrobials circulating in the private health care market with little regulation. The current system of privatized and unregulated production and distribution of antibiotics is largely obscured from view and shielded from critical inquiries as an important contributing factor for overuse of antimicrobials.

The second strategy implicitly assumes that the selective pressure that gives rise to resistant strains is confined to drugs that can kill microbes directly and not to other medical interventions like vaccines or therapeutic antibodies; it is a view that obscures the inherent interdependence of humans and microbes, which would predict adaptive microbial mutations to arise in response to any selective pressure on the human–microbe interactions. Therapeutic antibodies are a relatively new phenomenon, with their application thus far limited to specific populations because of their high prices, and they have not resulted in emergence of clinical resistance. Whether this observation would hold when it is deployed at a global scale remains to be seen.

In short, the dominant policy responses implicitly externalize the cause of AMR outside the current system governing the human production and use of antimicrobials. The One Health framework is now mystified as “complex and not amenable to change, and further ridden with implementation challenges” to be circumvented with simple technologies [20,53,59,60]. The material reality underlying the production and distribution of antimicrobials, including the rules permissive to large-scale commercial production, unregulated distribution through the market, and the consumption and disposal of antimicrobials is made invisible and continually evades critical inquiries.

#### 2.2.3. Summary

Despite the explicit call of the One Health framework to recognize AMR as an anthropogenic problem linked to the current mode of production and consumption of antibiotics, the dominant policy recommendations focus on individual behavior change and the development of next generation antimicrobials and alternative products to antimicrobials. The narrative portrays the problem of AMR as caused by microbes as an external threat detached from the political economy surrounding the production and use of antibiotics. It hides from view the current system governing the production and use of antibiotics as a source of the problem. Consequently, policies and programs have been limited to developing new antimicrobials since the 1930s. Despite the significant contribution made by antimicrobials in improving population health, AMR has been a recurrent problem since its first documented case in the late 1930s.

### 2.3. Case Study 3: Health Inequities in Racialized Communities

#### 2.3.1. Recurrent Nature of the Problem

Racial disparities in health outcomes have been reported for decades in multiracial countries including the USA [61,62], South Africa [63,64], UK [65], Australia [66], Brazil [67], and Canada [68]. For example, a report from 1950 describes the persistent disparities in health outcomes between the Native Americans and the rest of the Americans, including deaths due to TB, pneumonia, and typhoid. The report levels a scathing charge against the U.S. government for its failure to fulfill its responsibility to provide adequate health services to the Native Americans [61]. Similarly, African Americans have shown a higher risk of developing infectious diseases (e.g., AIDS) as well as non-communicable illnesses, such as diabetes and cardiovascular diseases [69,70].

**Figure 3:**
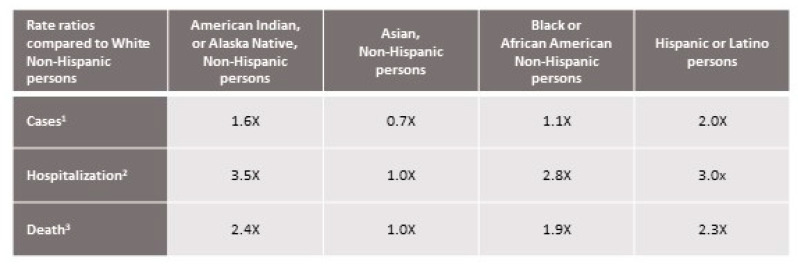
Risk for COVID-19 infection, hospitalization, and death by race/ethnicity [72].^1^ Data Source: Data reported by state and territorial jurisdictions (accessed 11 April 2021). Numbers are ratios of age-adjusted rates standardized to the 2019 U.S. intercensal population estimate. Calculations used only the 61% of case reports that have race and ethnicity data available; this can result in inaccurate estimates of the relative risk among groups. ^2^ Data Source: COVID-NET (https://www.cdc.gov/coronavirus/2019-ncov/covid-data/covid-net/purpose-methods.html, accessed 1 March 2020, through 3 April 2021). Numbers are ratios of age-adjusted rates standardized to the 2019 US standard COVID-NET catchment population. ^3^ Data Source: National Center for Health Statistics (NCHS) provisional death counts (https://data.cdc.gov/NCHS/Provisional-DeathCounts-for-Coronavirus-Disease-C/pj7m-y5uh, data through 3 April 2021). Numbers are ratios of age-adjusted rates standardized to the 2019 US intercensal population estimate. Reproduced with permission from the U.S. Center for Disease Control (accessed 18 April 2021) [72].

The enduring nature of health inequities in racialized communities has been exposed most recently during the COVID-19 pandemic. According to reports by the U.S. Center for Disease Control, Black or African Americans, Americans of Hispanic origin, and Native Americans and Alaska Natives are at increased risks of hospitalization and death from COVID-19 (Figure 3) [71,72]. Furthermore, the gap in life expectancy between non-Hispanic Black and white populations increased by 46% between 2019 and the first half of 2020 (from 4.1 to 6.0 years)—the largest gap since 1998 [73].

In the UK, people of African Caribbean or other Black ethnicities and the people of Pakistani, Bangladeshi, or Indian ethnicities have shown a significantly increased risk of death involving COVID-19 compared to those of the White ethnicity. People of Black ethnicities showed the highest risk of death, with approximately a three-fold greater risk compared to that shown for the White ethnicity group after adjusting for the age structure and the size of the population [74].

#### 2.3.2. Implicit Externalization the Cause

The predominant approaches to identify the underlying causes of health inequities among racialized communities are grounded in the framework of social determinants of health (SDOH), which acknowledges the importance of political, social, and economic forces that determine the circumstances in which people grow, live, work, and age [75]. For example, in the UK, the increased risk of COVID-19-related death for the people of Black ethnicities was found to be largely explained by socioeconomic factors measured by five sets of variables: (1) place of residence; (2) the Index of Multiple Deprivation, which represents an overall measure of deprivation based on factors such as income, employment, education, living environment, and access to housing; (3) household composition; (4) socioeconomic classification based on employment relations and occupations (e.g., professional managerial class) of the household head; and (5) self-reported health [76].

Similarly, the U.S. reports have associated the increased risks of COVID-19 disease and deaths among Black Americans and Latinx communities with poverty, poor access to healthy foods, lack of adequate health services and education facilities, poor living conditions, and high prevalence of clinical risk factors (e.g., diabetes, chronic cardiovascular disorders) [71,77,78]. Moreover, these communities tend to be engaged in low-wage jobs, and are over-represented in the service industry including restaurants, food processing, and early care and education, all of which are considered to be ‘essential work’ for which physical distancing is not an option [71]. Compounding these conditions further, only 30% of the bottom 10% wage earners in the U.S. have access to paid sick leave compared to 93% of the top 10% wage earners [71].

These reports highlight that health inequities observed in the racialized communities are rooted in inequalities in material conditions among these communities. What these reports fail to convey, however, is the relational character in the inequalities in material conditions. Inequalities by definition are *relational*, not individual. In capitalist material relations, conditions that concentrate resources in the privileged are the very conditions that permit deprivation of the marginalized. In these relational terms, deprivation of the marginalized is a necessary consequence, not an accident, of a system contingent on the concentration of wealth and power. In other words, inequalities in material conditions are an inner necessity of material relations in the capitalist mode of production and ownership.

Yet, this relational character in inequalities of material conditions, and hence, health equities, is removed from view and critical inquiries. The dominant narrative implicitly locates the cause of and solutions to the problem outside the material relations between the privileged and the marginalized. Consistent with the implicit externalization of the cause, the relational character of inequalities in wealth is kept invisible in the recommended solutions to solve the racial disparities in health. For example, in the context of COVID-19, the main recommendations include (1) education and training on how to recognize and modify implicit racist biases, (2) more material and personnel resources to improve access to and quality of care in the ‘underserved’ communities (e.g., provision of free COVID-19 testing centers, cost-free temporary housing to isolate non-critically ill and asymptomatic cases), and (3) educating the communities with excess mortality about the risks and preventative measures [71,77,78].

Provision of more resources and health facilities in the marginalized communities is necessary, but insufficient to solve health inequities. None of these recommendations recognizes the inherent tension between the privileged and the marginalized as a structural necessity of the material relations. Within this frame, the material relations that are predicated on inequalities are obscured and precluded from critical inquiries as a source of health inequities, and can continue unperturbed.

#### 2.3.3. Summary

Health inequities in racialized communities are rooted in the inequalities in wealth and power that are legitimized by racialization. The deprivation of health among racialized communities is directly related to the deprivation of material wealth among them. Within capitalist material relations, deprivation of resources from the marginalized is a necessary consequence of concentrating resources within the privileged. In other words, racial disparities in wealth and health are rooted in the material relations of our economic system, not in the malfunctioning of the system. The dominant narrative, however, forecloses the material relations inherent in our economic system as a cause of the health inequities among racialized communities. It implicitly seeks causes of and solutions to racial inequities in health outside the material relations embedded in the system as if it is an accident. Rules that permit extreme concentration of wealth for the privileged and deprivation of it for the marginalized evade scrutiny and remain unperturbed. As a result, health inequities among racialized communities have been a recurrent problem in multiracial societies with economic inequalities.

## 3. Conclusions

Ideology and our inability to observe and critique ideology remain a formidable barrier to solving global health inequities that are rooted in social inequities. While systematic approaches to identify and critique ideological operations in health programs would be valuable, there has been little research on theoretical frameworks to guide such efforts.

In this paper, we synthesized what may be considered symptoms of ideological operation based on Žižek’s conceptualization of ideology, and we illustrated an approach to identify ideological operation by using three case studies of recurrent global health problems. It is not the objective of this article to present an analysis or formal proof of the causes of the three global health problems illustrated, which are necessarily multifactorial. Our central argument is that the dominant discourse implicitly forecloses the analysis of the social structure itself as a source of the problem. It is precisely in this foreclosing that we identify symptoms of an ideological practice, which limits our understanding of the problems and possibilities for solving them.

The recurrent failure to solve inequitable access to vaccines, AMR, and health inequities in racialized communities can be interpreted as a consequence of ideological practice that limits us to cosmetic changes, while keeping the root causes undisturbed. In concrete terms, our analysis reveals areas of further research and action that are otherwise non-visible and precluded in the dominant discourse.

For example, confronting the inherent contradiction between the desire to provide vaccines as a public good and the rules of the production and ownership of vaccines as a commodity can illuminate new directions for research and action on different approaches to increase public control and ownership of public goods like vaccines. Such research would guide us to re-imagine relations among national governments, non-government/non-profit actors, and commercial vaccine manufacturers. It would illuminate new mechanisms to support vaccine production and distribution at the national and regional levels to ensure an equitable provision of vaccines globally based on transnational solidarity, and to avoid vaccine nationalism.

Similarly, if we confront the cause of AMR within the relation between humans and microbes, which encompasses the human production and consumption of antibiotics, rather than conceptualizing microbes as a threat to an otherwise harmonious hierarchy between humans and microbes, we would be freed from the limited search for solutions within the race between microbes and antimicrobials. We can imagine new approaches to ensure the survival and wellbeing of the human species within the dynamic and chaotic ecosystem in which we constantly interact with and influence other organisms. In the re-imagined frame, technological solutions to specifically target microbes, like vaccines and antimicrobials, would merely be one of many approaches, but not the dominant approach that we rely on, to ensure healthy individuals and healthy communities.

In the case of the recurrent failure to solve health inequities in racialized communities globally, locating the causes of the problem within, and not outside, capitalist material relations would illuminate areas of further research and action around the rules governing generation and ownership of resources across social groups, relations of power that determine such rules, and social mechanisms that implicitly legitimize unequal and unjust relations of power and wealth, such as racialization and the neoliberal narrative of meritocracy [14,19].

We believe that our new, generalized criteria for identifying ideology allows one to identify ideological practices that could not be identified by particular ideological contents. It also liberates us from an illusory search for emancipatory content. Critiquing ideology in general reveals possibilities that are otherwise kept invisible and unimaginable, and may help us solve recalcitrant problems like health inequities.
